# Mechanistic Characterization and Molecular Modeling of Hepatitis B Virus Polymerase Resistance to Entecavir

**DOI:** 10.1371/journal.pone.0009195

**Published:** 2010-02-12

**Authors:** Ann W. Walsh, David R. Langley, Richard J. Colonno, Daniel J. Tenney

**Affiliations:** Research and Development, Bristol-Myers Squibb Inc., Wallingford, Connecticut, United States of America; Saint Louis University, United States of America

## Abstract

**Background:**

Entecavir (ETV) is a deoxyguanosine analog competitive inhibitor of hepatitis B virus (HBV) polymerase that exhibits delayed chain termination of HBV DNA. A high barrier to entecavir-resistance (ETVr) is observed clinically, likely due to its potency and a requirement for multiple resistance changes to overcome suppression. Changes in the HBV polymerase reverse-transcriptase (RT) domain involve lamivudine-resistance (LVDr) substitutions in the conserved YMDD motif (M204V/I ± L180M), plus an additional ETV-specific change at residues T184, S202 or M250. These substitutions surround the putative dNTP binding site or primer grip regions of the HBV RT.

**Methods/Principal Findings:**

To determine the mechanistic basis for ETVr, wildtype, lamivudine-resistant (M204V, L180M) and ETVr HBVs were studied using in vitro RT enzyme and cell culture assays, as well as molecular modeling. Resistance substitutions significantly reduced ETV incorporation and chain termination in HBV DNA and increased the ETV-TP inhibition constant (K_i_) for HBV RT. Resistant HBVs exhibited impaired replication in culture and reduced enzyme activity (k_cat_) *in vitro*. Molecular modeling of the HBV RT suggested that ETVr residue T184 was adjacent to and stabilized S202 within the LVDr YMDD loop. ETVr arose through steric changes at T184 or S202 or by disruption of hydrogen-bonding between the two, both of which repositioned the loop and reduced the ETV-triphosphate (ETV-TP) binding pocket. In contrast to T184 and S202 changes, ETVr at primer grip residue M250 was observed during RNA-directed DNA synthesis only. Experimentally, M250 changes also impacted the dNTP-binding site. Modeling suggested a novel mechanism for M250 resistance, whereby repositioning of the primer-template component of the dNTP-binding site shifted the ETV-TP binding pocket. No structural data are available to confirm the HBV RT modeling, however, results were consistent with phenotypic analysis of comprehensive substitutions of each ETVr position.

**Conclusions:**

Altogether, ETVr occurred through exclusion of ETV-TP from the dNTP-binding site, through different, novel mechanisms that involved lamivudine-resistance, ETV-specific substitutions, and the primer-template.

## Introduction

Approximately 350 million people worldwide are chronically infected with hepatitis B virus (HBV) [1]. HBV is the most prevalent chronic liver infection, often leading to cirrhosis, liver failure and primary hepatocellular carcinoma [reviewed in [2]] and responsible for nearly one million deaths per year [1]. Treatments for chronic HBV include parenteral regimens containing the immunomodulator interferon α or oral nucleoside/nucleotide analogue reverse transcriptase inhibitors (NRTI) which target the reverse transcriptase (RT) activity of the HBV polymerase, the only enzymatic target of the virus. Five NRTIs are approved for treating chronic HBV in the U.S., including lamivudine (LVD), the phosphonate adefovir, administered as a dipivoxil prodrug, telbivudine, the phosphonate tenofovir, administered as a disoproxil fumarate prodrug, and entecavir (ETV).

The NRTI inhibitors approved for HBV therapy can be described structurally as having L-configuration ribose isosteres of LVD and telbivudine, alkyl chain ribose isosteres of adefovir and tenofovir, or, in the case of ETV, a D-configuration cyclopentyl ribose isostere with an exocyclic alkene replacing the ribose furanose oxygen. We previously reported that the novel structure of ETV can be modeled into a unique hydrophobic pocket within the HBV RT dNTP binding site, consistent with the observed potency of ETV and activity against HBV with resistance substitutions to either the L-nucleoside analogs or alkyl chain phosphonates [3]. ETV HBV DNA chain termination was also found to occur by a novel mechanism [3], [4]. The prior modeling studies also revealed the basis for partial cross-resistance of LVDr HBV to ETV since the pocket accessed by ETV was partly reduced in HBV with LVDr substitutions of the tyrosine-methionine-aspartate-aspartate (YMDD) dNTP-binding active site loop of the polymerase [3].

HBV resistance to ETV was initially identified in two Phase II clinical study patients with LVDr HBV that acquired additional substitutions and exhibited virologic breakthrough during ETV therapy [5]. Both patients were infected with HBV with M204V+L180M LVDr substitutions at study entry and developed additional substitutions T184G & S202I, or M250V on therapy. Breakthrough isolates displayed high levels of phenotypic resistance to ETV *in vitro*. The T184, S202 or M250 ETV signature substitutions did not display significant resistance in the absence of LVDr changes, establishing that multiple substitutions were required for phenotypic resistance leading to virologic breakthrough on ETV. Resistance studies of patients treated for up to 5 years with ETV have shown that ETV-resistance (ETVr) is rare (∼1% over 5 Years) in nucleoside naive patients, consistent with a high barrier to resistance [6]. Resistance occurs more frequently in ETV patients with LVDr HBV, due to the requirement for only one additional substitution at T184, S202 or M250 for high level ETVr. However, different ETVr substitutions result in various levels of phenotypic ETVr [7], [8]. In addition, ETVr substitutions in isolates with only the M204I substituted LVDr HBV and not the M204V+L180M LVDr virus, phenotypic resistance to ETV is diminished.

In this report we use cell culture, *in vitro* enzyme and molecular modeling studies to characterize the mechanism(s) for phenotypic ETVr in HBV with signature ETVr substitutions at T184, S202 or M250 in LVDr HBV. The results show that ETVr substitutions in LVDr HBV cause further restriction of the ETV-triphosphate (ETV-TP) binding pocket, through steric influences but also by disrupting stabilizing interactions of the active site YMDD loop. In addition, ETVr substitutions in the primer grip region of HBV RT appear to operate through an additional novel mechanism as revealed by template-dependent phenotypic changes and effects on activity through the nucleotide-binding pocket. The results also suggest the basis for a lack of ETVr in HBV lacking LVDr substitutions.

## Results

### ETV Resistance (ETVr)

HBV containing LVDr substitutions M204V+L180M exhibit reduced ETV susceptibility that can lead to virologic breakthrough with additional substitutions at ETVr signature residues T184, S202 or M250 [5], [8]. The levels of ETVr observed in cell culture and *in vitro* enzymatic assays of representative clones with ETVr substitutions are shown in Table 1. ETV and ETV-TP displayed potent inhibition of wildtype HBV in culture and HBV polymerase RT *in vitro* with EC_50_ and IC_50_ values of 3 and 0.5 nM, respectively. LVDr HBV shows approximately 8-fold cross-resistance to ETV in culture [3], [7], despite higher fold changes for LVDr HBV RT *in vitro*, likely a result of efficient intracellular phosphorylation to the triphosphate at nanomolar exposures [9]. High levels of ETVr were observed when HBV clones contained ETVr signature substitutions in a LVDr background both *in vitro* (>400-fold) and in culture (>200-fold), respectively (Table 1). A full analysis of the different ETVr substitutions in culture has been reported for engineered [8] and clinical [7] ETVr HBV isolates. A previously described isolate containing both T184G and S202I substitutions [5] displayed the highest ETVr levels, suggesting that multiple ETVr changes in a LVDr background may have additive or synergistic effects. Resistance was specific for ETV-TP as the IC_50_ values for the same ETVr isolates against 2′,3′-dideoxyguanosine-5′-triphosphate (ddGTP), a known competitive inhibitor of HBV RT, all fall within a similar range of approximately two-fold above background (Table 1). The cross-resistance profile for these ETVr substitutions has been shown to be relatively restricted in cell culture, as ETVr HBV does not show reduced susceptibility to adefovir [5], [10] or tenofovir [11].

**Table 1:** Phenotypic ETV Resistance of HBV Clones.

	Cell Culture	*In Vitro* Polymerase	*In Vitro* Polymerase
	ETV EC_50,_ nM, Average ± SD (Range)^a^	ETV-TP IC_50_, nM, Average ± SD (Range)^a^	ddGTP IC_50_, nM, Average ± SD (Range)^a^
**Wildtype** b	3.0±1.5 (1.4–5.2)	0.5±0.1 (0.4–0.7)	87.6±8.4 (78.0–92.9)
**LVDr L180M + M204V** c	30.4±21.6 (10.4–58.6)	27.1±5.6 (20.9–31.9)	137.9±10.0 (123.5–148.3)
**LVDr + T184L** d	738.4±119.4 (654.0–822.8)	372.9±142.7 (236.4–542.8)	ND
**LVDr + S202G** d	1,207.3±1,051.6 (463.7–1,950.9)	258.3±25.5 (222.7–290.8)	125.1±8.8 (120.0–135.2)
**LVDr + M250V** d	3,083.6±1,795.8 (1,813.7–4,353.4)	229.0±49.5 (192–323.9)	153.7±51.4 (117.3–190.0)
**LVDr + T184G + S202I** e	>4,000 (>4,000)	636.1±19 (617.2–655.2)	187.6±6.7 (180.0–192.7)

a EC_50_ and IC_50_ values represent the mean ± standard deviation (SD) from at least 3 independent experiments.

b Wildtype values from a single isolate tested in parallel [5], the average ETV EC_50_ for a panel of 76 wildtype isolates  = 3.4±2.0 nM.

c LVDr values from a single isolate tested in parallel [5], the average ETV EC_50_ for a panel of 15 LVDr isolates  = 31.1±17.5 nM.

d Values averaged from 2 independent isolates.

e Values obtained from a single patient isolate [5].

### Kinetic Studies

To explore the mechanism(s) underlying ETVr, we compared enzymatic parameters for the wildtype and resistant enzymes. Detailed kinetic studies for the HBV polymerase were not possible due to the limitations of studying nucleocapsid-associated enzyme covalently linked to its minus strand DNA template. Nevertheless, important mechanistic information was gained. The recognition for the natural dGTP substrate was only modestly affected by ETVr changes, which increased K_m_ values 2.2- to 2.6-fold (Table 2). The only exception was the most resistant, quadruple-substituted RT with ETVr T184G+S202I changes in a M204V+L180M LVDr background, where the apparent K_m_ for dGTP was approximately 7-fold less than that for wildtype. The calculated K_i_/K_m_ ratios revealed that the wildtype HBV RT preferred ETV-TP over dGTP [K_i_/K_m_ ratio <1], as previously reported [4], [9]. The LVDr HBV polymerase showed a modest (30-fold) alteration in the K_i_ for ETV-TP relative to the striking (>300 to >500-fold) changes exhibited by the ETVr polymerases. The resulting high K_i_/dGTP K_m_ ratios for the ETVr enzymes suggested preferential recognition of dGTP relative to ETV-TP, more than 600-fold changed from the wildtype.

**Table 2:** Kinetic Parameters for HBV Polymerases.

	dGTP K_m_, nM^a^ (Fold WT)^b^	K_i_, nM (Fold WT)	K_i_/K_m_ (Fold WT)	k_cat_, min^−1^ (Fold WT)
**Wildtype (WT)**	5.0±0.5 (1)	0.4 (1)	0.08 (1)	0.24 (1)
**LVDr M204V+L180M**	3.5±0.4 (14)	12.3 (30.8)	3.5 (43.8)	0.093 (2.58)
**LVDr + T184L**	1.9±0.6 (2.6)	224.5 (561.3)	118.2 (1477.5)	0.13 (1.85)
**LVDr + S202G**	2.1±0.1 (2.4)	146.7 (366.8)	69.9 (873.8)	0.11 (2.18)
**LVDr + M250V**	2.3±0.5 (2.2)	121.2 (303.0)	52.7 (658.8)	0.17 (1.41)
**LVDr + T184G + S202I**	0.7±0.1 (7.1)	155.1 (387.8)	221.6 (2770.0)	0.076 (3.16)

a Values are the mean ± standard deviation from at least three independent experiments.

b Fold change from wildtype value.

In agreement with other reports, HBV with LVDr substitutions exhibited impaired HBV replication in culture [12], [13], [14]. HBV isolates with ETVr substitutions were also shown to be replication impaired in culture [5], [8]. To determine if ETVr substitutions also negatively affected HBV polymerase catalytic efficiency, we determined the estimated k_cat_ or turnover number as a measure of the number of reaction complexes that could be converted to product, per molecule of enzyme per unit of time. As illustrated in Table 2, all resistant polymerases displayed reductions in enzymatic activity relative to the wildtype, with the ETVr quadruple mutant showing the greatest reduction (3.7-fold less than the wildtype). The reduced polymerase activity of the ETVr enzymes is consistent with reduced replication of the resistant viruses in culture.

### ETVr HBV Incorporate Reduced Levels of ETV

**Figure 1:**
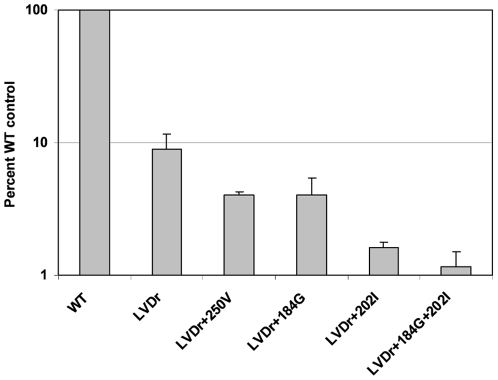
Incorporation of [^3^H]-ETV into HBV nucleocapsid DNA in culture. [^3^H]-ETV was added to cultures of HepG2 cells transfected with an HBV expression construct, as detailed under Materials and Methods. HBV nucleocapsids were isolated from cell lysates, as detailed under Materials and Methods, and radiolabeled HBV DNA was quantified through scintillation counting. The levels of nucleocapsid-associated [^3^H] from cells grown in [^3^H]-ETV are presented as percent wildtype control values ± standard deviation. Yields of HBV nucleocapsid DNA were standardized according to real-time PCR quantification of HBV DNA within isolated nucleocapsids, as detailed under Materials and Methods. Similar results were obtained by standardizing total HBV nucleocapsid DNA levels with nucleocapsids from parallel cultures metabolically labeled with [^3^H]-thymidine (data not shown). WT, wildtype nucleocapsids; LVDr, M204V+L180M substituted HBV nucleocapsids, LVDr+M250V, M204V+L180M+M250V substituted nucleocapsids; LVDr+T184G+S202I, M204V+L180M+T184G+S202I substituted nucleocapsids. HBVs were tested independently 3 to 4 times, except the LVDr+M250V, which was tested twice.

The experiments above suggest ETVr substitutions reduce the recognition of ETV-TP by HBV polymerase. Since the proposed mechanism of activity for ETV involves addition to the growing HBV DNA followed by chain termination [3], HBV RTs with ETVr should less efficiently recognize and incorporate ETV. To test this, we determined the level of [^3^H]-ETV incorporated into the DNA of various HBVs in culture (Figure 1), relative to the total HBV DNA synthesized. To control for different levels of DNA produced by the various HBVs, the levels of HBV DNA in the nucleocapsid preparations were quantified according to HBV real-time PCR performed on the samples (Fig. 1), or [^3^H]-thymidine incorporation in a parallel sample (not shown). Scintillation counting and standardization revealed that ETVr HBV contained a lower percentage of the total nucleocapsid DNA radiolabeled with [^3^H]-ETV than the wildtype (Figure 1). LVDr HBV had an average of 9% [^3^H]-ETV radiolabeled DNA relative to the wildtype while the ETVr substitutions resulted in average levels from 1.2 to 4% of wildtype (Fig. 1). These results extend the kinetic studies by showing that the reduced recognition of ETV by ETVr RT was accompanied by reduced incorporation, and presumably, chain termination.

HBV polymerase has been shown to be capable of exonucleolytically proofreading chain terminating nucleotides from the 3′ end of DNA through pyrophosphorolysis [15]. While the results in Figure 1 do not exclude this mechanism of resistance, non-obligate chain terminating NRTIs such as ETV have been found to be resistant to pyrophosphorolysis [16] because DNA chain termination occurs 1 to 3 bases after the NRTI is incorporated. We have been unable to demonstrate removal of ETV incorporated into HBV DNA using pyrophosphorolysis with either wildtype or ETVr enzymes (data not shown). The inability of ETV to be proofread by HIV RT has been reported by Tchesnokov et al. [17].

### Molecular Modeling

**Figure 2:**
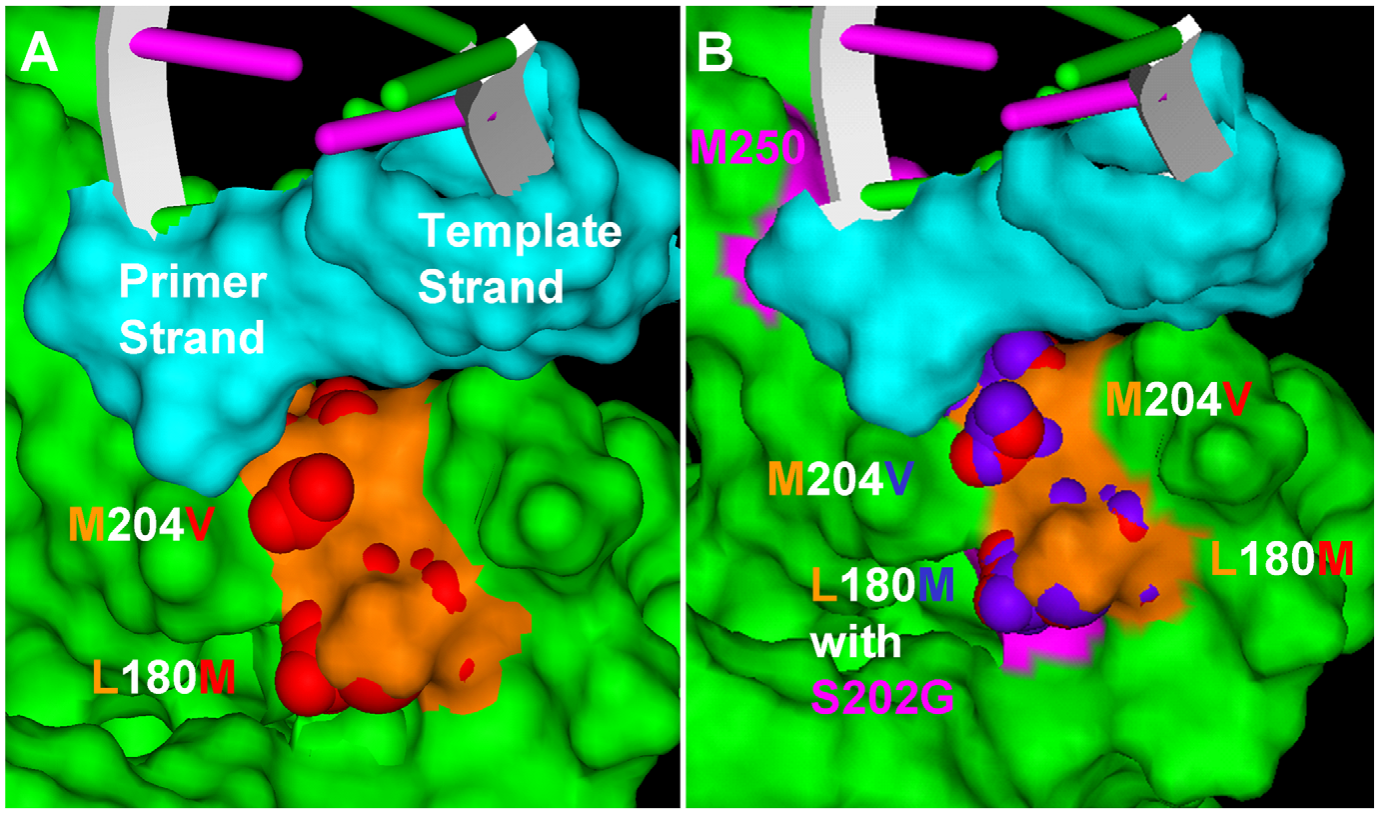
Molecular homology model of resistant HBV RTs. The ETV-TP binding pocket of HBV RT. [3]. (A) HBV RT with LVDr substitutions, M204V+L180M, (B) HBV RT with LVDr + S202G. HBV RT, ETV-TP and primer-template DNAs are labeled. The residues lining the pocket are orange, changes from LVDr are red, from ETVr are blue, and the M250, S202, and T184 residue positions (panel B) are pink. Panel A was essential reproduced from [3] with permission.

A previously described molecular model [3] was used to further explore the impact of the ETVr changes on the HBV RT. Studies using this homology model of HBV-RT/DNA/dGTP complex based on the HIV-1 RT/DNA structure revealed that the exocyclic alkene of ETV-TP fit into a hydrophobic pocket at the rear of the dNTP binding site of RT [3]. The LVDr substitutions M204V+L180M reduced the size of this pocket to exclude the binding of LVD-TP with its larger oxathiolane ring [18] but not the binding of ETV-TP [3]. The ETV binding pocket of LVDr HBV (M204V+L180M) is shown in Figure 2A. Several different ETVr substitutions have been observed in ETV-treated patients, including T184A/C/F/G/I/L/M/S, S202C/G/I, and M250 I/L/V. Cell culture phenotypic analysis has revealed that not all of these substitutions result in significant reductions in ETV phenotypic susceptibility [8].

### T184 and S202 Substitutions

Introduction of the ETVr signature substitutions into LVDr HBV RT caused further reduction of the size of the ETV-binding pocket to sterically impede ETV-TP binding, in agreement with the experimental findings above. Figure 2B shows that introduction of the S202G substitution causes repositioning of the L180M and M204V residues to further restrict the ETV-TP binding site. T184 substitutions caused similar changes to the LVDr HBV RT (not shown).

### Interaction of T184 and S202 Residues

**Figure 3:**
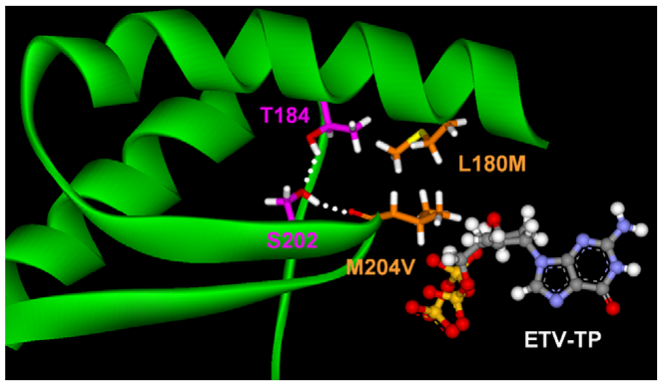
The T184-S202-204 hydrogen bonding network stabilizes the YMDD loop. The YMDD loop is shown with residues M204V+L180M and the H-bonding between residues T184, S202 and M204V are shown as dotted white lines.

The LVDr substitutions M204V and L180M lie juxtaposed in the HBV RT dNTP binding pocket, the M204 positioned within the YMDD active site loop and the L180 residue in the adjacent alpha helix. Interactions between these structural motifs were first proposed upon the double substitution M204V+L180M encoding LVDr [18]. The S202 residue is also located on the YMDD active site loop of the HBV RT, juxtaposed to the T184 residue on the adjacent alpha helix. It is therefore interesting that substitutions at T184 and S202 residues in LVDr HBV can result in ETVr. In the HBV RT model, T184 forms a hydrogen bond with the side-chain hydroxyl group of S202, which in turn hydrogen bonds with the backbone carbonyl of residue 204 (204 valine in Figure 3). This hydrogen bonding network stabilizes the YMDD loop to optimally anchor the incoming dNTP and the 3′ nucleotide of the growing DNA strand for the SN2 addition reaction. In addition, the hydrogen bonding network holds the small pocket at the back of the dNTP binding site open. The S202G, C or S202I substitutions lose the ability to hydrogen bond with T184 and M204. The smaller, more flexible S202G substitution destabilizes the YMDD loop and closes the ETV binding pocket in the back of the dNTP site (Fig. 2B). The S202I change sterically closes the pocket as this region of the loop moves to accommodate the larger side chain. The results of comprehensive phenotypic analysis of substitutions at positions S202 [8] indicated that S202A/G/C/T/V/N replicated well and S202 I/F/Y replicated poorly, but that other S202 changes did not replicate. Additionally, large substitutions S202F/I/Y and small substitutions S202G/A were highly resistant. These results are consistent with the model in that highly tolerated substitutions were uncharged, similar to serine in size, while resistant residues could be either large or small, which repositioned the YMDD loop closer or further from the stabilizing alpha-helix containing L180M, and could further impact the YMDD loop changes on the ETV-TP binding-pocket. Substitutions of larger and charged groups did not grow well [8]. This is likely due to a misfolding or positioning the YMDD loop into the dNTP-binding site. Residues similar in size to the serine grow well and were not resistant or only modestly resistant, while small residues (G,A) were highly resistant.[8]

The T184 substitutions can also be divided into two functional types. The smaller T184G substitution (relative to threonine) allows the YMDD loop to be more flexible which dynamically closes the ETV binding pocket. T184 A and S changes also fit this model. The larger substitution T184L forces the ETV binding pocket closed as the YMDD loop is repositioned to accommodate the larger amino acid. T184 I, M, F, substitutions also fit this pattern. Therefore ETVr substitutions at T184 or S202 can cause resistance to ETV through steric repositioning of the YMDD loop and shrinking the ETV-TP binding pocket, or by disrupting the H-bonding network that stabilized the active site conformation. The T184G substitution [5] was also modeled into the LVDr HBV RT and produced a similar change in the ETV-binding pocket as the S202G change in Figure 2 (not shown). The results of comprehensive analysis of changes at T184 [8] were explained by the modeling; only charged residues did not grow well[8]. Among those that grew well, both large (T184L/M/F/Q/N) and small (T184A/G/C) residues resulted in ETVr.

### M250 Substitutions

**Figure 4:**
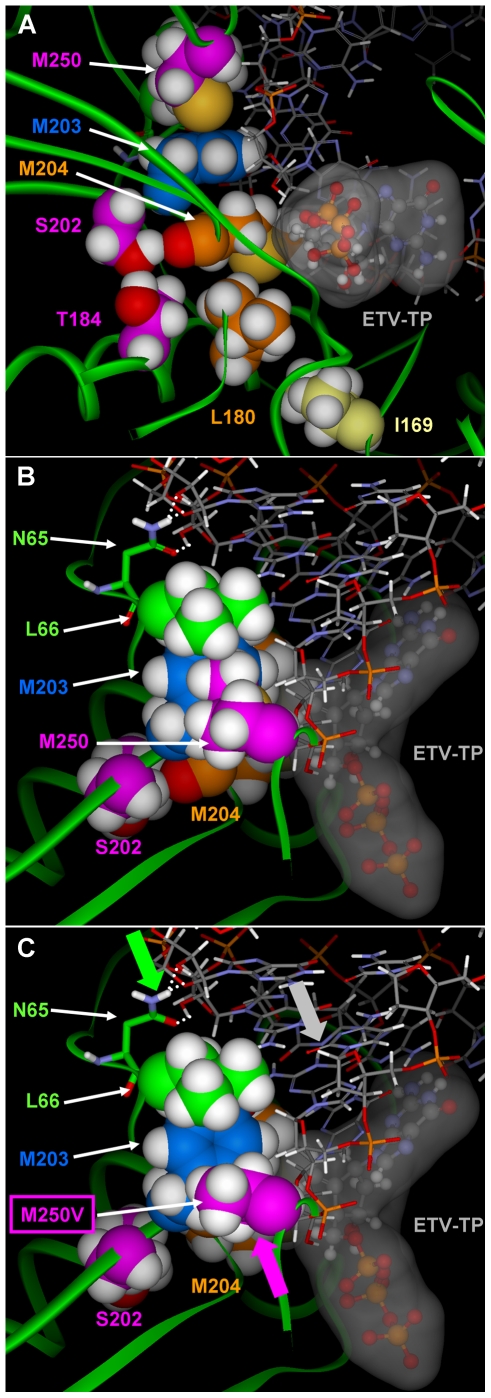
Position of residue M250 in the HBV RT/DNA molecular model. A) The relative location of the M250, Y203, M204V, and other resistance residues are shown with primer-template DNA and ETV-TP in the dNTP binding site. The proximity of the M250 residue to both primer DNA and the Y203 residue are shown, indicating possible mechanisms for effects of M250 substitutions on the dNTP binding site, as well as the influence different side chains could have on primer positioning. B) M250 is packed against L66 and N65 forms hydrogen bonds (white dotted lines) to the hydroxyl groups on the backbone of the RNA template strand. C) The smaller side chain of the M250V no longer packs against L66. As indicated by the arrows (protein:green; DNA:gray; M250V:purple), the resulting conformational change in the protein to close the resultant hole repositions the RNA/DNA slightly over the NTP binding site. The slight modification to the NTP site enhances LVD binding while decreasing the binding affinity of ETV.

Substitutions at residue M250 within LVDr HBV that result in ETVr are less straightforward to interpret and suggest resistance may be imparted by changes in both the dNTP binding site and in the position of the growing DNA chain. In our model, the M250 residue lies in the primer grip region of the HBV RT [18] and the methionine side chain packs against Y203 of the YMDD loop, the dNMP at the +2 position of the primer DNA strand, and L66 (Figure 4). In our previous study [3] it was proposed that ETV chain termination resulted from steric contact between ETV and Y203 and L66. Therefore, substitution of M250 with a shorter, branched valine or leucine could mitigate HBV DNA chain termination of ETV adduct by providing room for Y203 and L66 to move out of the way as ETV moves through the +1, +2, and +3 position. However, the lack of increased [^3^H]-ETV incorporation into HBV DNA (Figure 1) does not support readthrough as a resistance mechanism. Alternatively, in the model of M250V, the valine side chain packs tightly against the dNMP at the +2 position and produces a small hole between M250V/L and Y203 and L66. Amino acid residues K60-L66 pack against the RNA or DNA ribose backbone of the template strand and N65 simultaneously hydrogen bonds with two adjacent 2′-hydroxyl groups when the template strand is RNA. In response to the M250 mutation, the DNA slightly pivots in the active site to relieve the steric compression between M250V and the +2 dNMP and L66/N65 as they move to fill the hole between M250V and Y203 and L66. This slightly repositions the primer strand over the ETV-TP/LVD-TP binding pocket, subtly changing the shape of the pocket within the dNTP binding site. This results in an increase in the size of the pocket near M204V and shrinking of the corner of the pocket comprised by the dNMP in the +1 position of the template strand. The pivoting of the nucleic acid duplex is amplified due to the additional 2′-hydroxyl groups on the template strand of the DNA/RNA complex. Comprehensive phenotypic analysis of M250 substitutions in LVDr HBV [8] provided support for the model: Again charged residues did not grow well[8]. This is consistent with the hydrophobic packing of M250 against L66 (Figure 4). M250 substitutions that were smaller than methionine and replicated well, M250A/G/S/T/C/D/E/Q/N/V/L/I, were all resistant. Those that were large, replicated and could maintain the positioning of the primer:template, M250F/Y, retained ETV susceptibility. In the model, the M250 substitutions F and Y provided edge to face stacking with Y203 and packed against L66. Altogether, these results are consistent with the model that the primer-grip positioning of the primer template modified the ETV-TP pocket.

### Experimental Evidence for the Mechanism of M250-Dependent Resistance

Experimental results suggest M250 changes affect the dNTP binding pocket. The M250V substituted HBV RT in the absence of LVDr changes was constructed and tested for susceptibility to LVD, an obligate chain terminator which should not be impacted by the mechanism that could potentially affect ETV chain termination. Replication of the singly M250V substituted HBV was 7-fold *hyper*sensitive to LVD in culture (wildtype HBV LVD EC_50_ = 794 nM, M250V HBV LVD EC_50_ = 116 nM), suggesting the M250 changes affected the dNTP binding site and not just elongation of the growing DNA.

**Figure 5:**
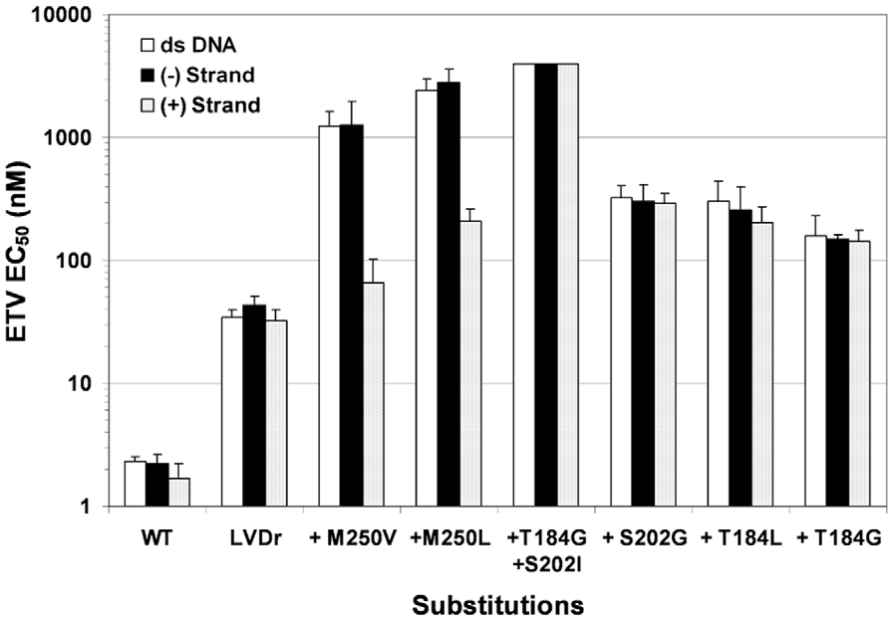
Analysis of ETVr through strand-specific DNA synthesis in culture. Cell culture ETV EC_50_ determinations were made for wildtype and various resistant HBVs. The levels of HBV DNA synthesized in the presence of ETV was determined by HBV-specific probe hybridization to HBV nucleocapsid DNA from the cultures. The probes used were the typical double-stranded DNA probe, or strand-specific riboprobes which hybridized to a single HBV DNA strand. Comparison of strand-specific EC_50_ versus double stranded EC_50_ for wildtype polymerase (WT) or LVDr M204V+L180M HBV, or the LVDr substitutions with ETVr substitutions (+T184, +S202, +M250) as indicated. Values at 4000 nM indicate that 50% inhibition was not observed at the highest ETV concentration tested.

Another set of experiments showed that the M250 substitutions in LVDr HBV decreased ETV susceptibility in a template-dependent manner. ETV inhibits HBV polymerase synthesis of DNA *in vitro* during both RNA-directed first (minus) strand synthesis as well as DNA-directed second (plus) strand synthesis [4]. To determine if ETVr was influenced by the template for DNA synthesis, cell culture EC_50_ determinations were made using strand-specific riboprobes to hybridize to the synthesized HBV DNA, in parallel with the typical double-stranded DNA probe. In this way an ETV EC_50_ for each strand of DNA synthesis during the HBV cell culture was determined. The EC_50_ determinations using the strand-specific DNA hybridization showed that HBV nucleocapsids containing wildtype, LVDr substituted and LVDr + T184 or S202 substitutions displayed nearly equivalent levels of susceptibility (EC_50_) in synthesis of both stands of HBV DNA (Figure 5, compare each strand probe to the results using the double-stranded DNA probe). In contrast, ETVr due to substitutions of M250V or M250L in LVDr HBV was found to be manifested primarily during RNA-directed minus strand DNA synthesis, as the EC_50_ for the first (minus) strand was increased relative to that for the second (plus) strand. The minus strand EC_50_ was similar to the EC_50_ obtained using the double-stranded DNA probe. These observations are consistent with M250 changes in the primer-grip region of HBV RT influencing ETVr at least in part through the template for DNA synthesis. An independent observation supported this hypothesis; resistance was seen using nucleocapsids prepared in our typical manner (Materials & Methods), isolated from cells grown in PFA to inhibit minus strand synthesis, enhancing the in vitro minus-strand synthesis activity of the preparations [19]. If experiments were performed using nucleocapsids from cultures without PFA, or if enzyme reactions were extended from the standard 1 hour to 3 hours in duration, both conditions that favored plus-strand DNA synthesis, the cell culture resistance to ETV of the LVDr+M250V virus was not observed enzymatically *in vitro* (data not shown). In contrast, substitutions at T184 or S202 in LVDr HBV resulted in ETVr under all these conditions. Thus, only M250 ETVr substitutions displayed template dependence.

## Discussion

The emergence of resistance is an intrinsic challenge with antiviral therapy. The nature and mechanisms of resistance for each therapy are important to develop treatment strategies, including for patients already harboring resistant viruses. Resistance to LVD can arise from a single substitution at residue M204 in the YMDD active site loop of HBV polymerase (with or without a compensatory change at L180) and results in high-level phenotypic resistance and clinical failure [20], [21], [22]. This YMDD substitution reduces the size of the LVD binding pocket, also causing complete cross resistance to other L-configuration ribose NRTIs such as telbivudine and emtricitabine [5], [10], [18], [23]. A similar profile is observed with YMDD changes in HIV RT [24], [25], [26], [27]. In contrast, resistance to adefovir leading to virologic breakthrough can arise from either A181 V or T substitutions [28], or N236T [29]. Modeling studies suggest that the N236T change may indirectly affect binding of the terminal γ-phosphate of adefovir-diphosphate [30], [31] and changes at A181 of the alpha helix adjacent to the YMDD active site loop likely affect the position of the YMDD loop itself or the nucleotide in the +1 position through interactions of A181V or T with the M204 S-methyl moiety of the YMDD loop [3]. A181 changes also impart LVDr [31], [32], [33].

The resistance pattern emerging from clinical studies of ETV suggest a more complex scenario; multiple changes in the HBV RT are required for clinically meaningful resistance, substitutions at T184, S202 or M250 in the HBV RT must be accompanied by LVDr substitutions M204V/I ± L180M [10]. However, high level phenotypic resistance does not occur when ETVr substitutions are present with only the M204I LVDr substitution [7] and does not result in virologic breakthrough [6].

In the present study, cell culture, *in vitro* enzyme, and molecular modeling approaches were used to show that the mechanism for ETVr involved decreased recognition and binding of ETV-TP by the resistant HBV polymerase. While ETV-TP accesses the same binding pocket in the HBV RT as LVD-TP, the exocyclic alkene group of the ETV-TP ribose isostere is small enough that binding is retained with LVDr changes while the larger oxathiolane moiety of LVD-TP can no longer be accommodated [3]. Further changes at positions T184, S202 or M250 in LVDr HBV, however, prevent ETV-TP binding. These ETVr signature substitutions in LVDr HBV RT resulted in substantial increases in ETV-TP K_i_ values. However, the majority of the ETVr RTs showed only a modest decrease in apparent K_m_ values for natural dGTP. Consistent with this finding was the observation that little cross resistance was observed between the ETVr RTs and dideoxy-GTP. These results suggest that recognition of natural nucleotides was not markedly altered by the ETVr RT changes, similar to LVDr YMDD changes in HBV [18], [34] and HIV RTs [24].

Decreased recognition and utilization of ETV-TP by the resistant enzymes resulted in reduced intracellular incorporation of [^3^H]ETV-MP into HBV nucleocapsid DNA inside cells. Since the proposed mechanism for ETV activity involves incorporation into the growing DNA chain followed by chain termination a few bases downstream, these results highlight the ultimate effect of ETVr. The fact that phenotypic resistance to ETV was observed in both culture and *in vitro* RT assays confirmed that the mechanism did not involve intracellular phosphorylation and was localized to the HBV RT activity.

Similar to LVDr HBV substitutions in the dNTP-binding pocket [12], [13], [14], ETVr changes were accompanied by replication defects to approximately 40-60% of the wild-type, similar to LVDr HBV [5] [8]. Here the catalytic efficiency of the ETVr polymerases was also reduced relative to the wildtype, confirming and providing a basis for the impaired HBV replication observed. The turnover number (kcat) of the ETVr polymerases were all approximately 2–3 fold lower than wildtype with the most resistant polymerase harboring the most ETVr substitutions having the lowest value. Despite these modest changes, they are consistent with the observations from cell culture studies. Although ETVr could presumably also involve enhanced replication through adaptive changes, the fact that resistance was observed through reduced ETV utilization and that resistant HBV and polymerase did not have enhanced replicative capacity, argue against the adaptive mechanism for resistance.

HIV resistance to LVD results from discrimination of incorporation of LVD-TP (exclusion) by steric hindrance (reviewed by Sarafianos et. al.[35]). Recent realization that ETV displays weak activity against HIV RT has prompted studies of ETV-TP in assays using purified HIV RT and defined templates [17], [36]. These studies [17] confirmed the delayed mechanism of ETV chain termination, shown previously in HBV [3], [4], and supported our modeling studies [3] which proposed the mechanism to be similar to that of fixed conformation bases [16], [37]. Furthermore, studies showed that LVDr YMDD-mutant HIV displayed reduced recognition of ETV-TP [36]. It is interesting to note that the potencies of ETV (EC_50_) and ETV-TP (K_i_) are significantly reduced against HIV [36], [38] relative to HBV [5]. Thus YMDD-mutant HIV shows susceptibility levels near or above the cytotoxic levels of ETV [17], [38], [39]. Therefore additional substitutions analogous to the T184, S202 or M250 substitutions in HIV might not be selected or provide increased resistance in the HIV of HIV/HBV co-infected patients treated with ETV for HBV.

The HBV ETVr changes at T184, S202 or M250 had little effect on ETV susceptibility in the absence of LVDr changes [5] and, among isolates from >1000 patients sequenced as part of our resistance surveillance program [10], [40] and unpublished), none of the three ETVr-specific changes was found in HBV that lacked LVDr substitutions. The present modeling experiments suggest the basis for finding ETVr changes only in the context of LVDr HBV. The M204 LVDr residue is critical to the stability of the YMDD loop and the dNTP binding pocket. The ETVr changes can further displace the position of the YMDD loop only after it has been destabilized by the M204 change. Perhaps this is why only a few changes to M204 are tolerated [14] while it appears that several changes can be accommodated at positions of ETVr [6]. Indeed the variety of ETVr substitutions found in patients includes T184A, C, F, G, I, L, M or S, S202C, G or I, and M250 I, L or V. Not all of these substitutions result in significant reductions in ETV phenotypic susceptibility or lead to virologic breakthrough [8]. Experiments suggest that this results from insufficient levels of either resistance or replication capacity, or both, for some of the substitutions [8]. In addition, we suggest that subtle steric or dynamic changes in the ETV-binding site, or the conformation of the active RT/ETV-TP/template structure, could affect levels of resistance.

An unexpected finding was that there would be differences in the mechanism of resistance between the T184 and S202 substitutions and those at position M250. The changes at T184 and S202 act through the same mechanism, by repositioning the YMDD loop and affecting the size of the ETV-TP binding pocket. However, different substitutions can have effects both sterically, by stably affecting the position of the YMDD loop and the pocket, or dynamically, by interrupting the stabilizing H-bonding network between the YMDD and the adjacent alpha helix. The larger T184L and S202I substitutions are examples that sterically affect the YMDD loop position. Substitutions T184G and S202G however are examples where the major changes appear to involve disruption of the stabilizing H-bonding network between T184 and S202.

Substitutions of M250 that result in ETVr, presumably within the ‘primer grip’ portion of RT [18], are less straightforward to explain mechanistically. While these changes also reduce the binding of ETV-TP by HBV RT, modeling suggests they act through contacts with Y203 or the primer strand of DNA. What we have seen is that M250 substitutions are unique in that resistance appears to reside primarily in minus strand DNA synthesis, suggesting that contacts with the primer-template may be involved in resistance. Indeed, during minus-strand synthesis, when the template is RNA, resistance at M250 is more apparent than when the template for synthesis is the narrower DNA template of plus-strand synthesis. Consistent with this theory is the finding that M250 substitutions with extended side chains, tryptophan, tyrosine and phenylalanine, retain ETV susceptibility. These residues likely can retain the positioning of the primer template, while those with smaller side chains do not, resulting in reduced ETV susceptibility [8].

There are still several unanswered issues concerning ETVr HBV. Firstly, why so many different substitutions are found at positions T184 and S202. Perhaps the basis lies in the fact that resistance can result from two pathways, either sterically with large residue substitutions, or dynamically by disrupting the H-bonding network in the active site. The fact that the overlapping HB surface antigen open reading frame is affected by changes at these positions may serve to restrict the repertoire of residues from a potentially larger list.

Additionally, genotypic HBV analysis of the patients with ETVr emerging on therapy during 5 year surveillance[6] revealed that 89% of ETVr patients had substitutions at T184, S202 or both, with 17% being a combination of the two. In contrast, only 11% had substitutions at M250, alone (5.5%) or in combination with those at T184 or S202 (5.5%). These results suggest that the M250 resistance substitution was much less common than those at T184 and S202, and even the combination of T184 and S202. This is surprising when considering that T184 and S202 changes can impact the sequence of the overlapping HBV surface antigen gene while those at M250 do not, as well as the fact that the vast majority of engineered changes at M250 resulted in phenotypic resistance to ETV [8].

An additional unanswered issue is why ETVr substitutions leading to virologic breakthrough occur only in LVDr HBV with the M204V+L180M substitutions and not M204I HBV [10]. We also have seen a very limited set of substitutions at T184 or M250 substitutions in viruses with the M204I LVDr change, and those that occur in both LVDr types show higher levels of resistance in the M204V+L180M background [7]. Our current hypothesis is that interactions between residues at position 204 and those at 184, 202 or 250 are important for a functional HBV RT enzyme, or perhaps that the ETVr substitutions further decrease the replication capacity of the more impaired M204I virus to below levels required to survive.

Finally, the impact of substitutions at residue I169 to ETVr must also be explored. I169T substitutions were found in both patients initially identified with ETVr [5] and various I169 substitutions have been seen in twelve of our other ETVr patients [6]. In general, several residues at position 169 were observed, including I169 L, M, T and S. In these patients, I169 substitutions were only observed to emerge coincident with or after primary ETVr substitutions at T184, S202 or M250, except in the case of a single LVD-treated patient. I169 changes were not required for virologic breakthrough or high level phenotypic resistance. I169 changes alone in LVDr HBV do not impart high level phenotypic ETV resistance, whereas changes at T184, S202 and M250 can. These observations are all consistent with the I169 substitution being a secondary or ancillary substitution for ETVr, perhaps fulfilling an adaptive change to compensate for replication impairment imparted by the primary ETVr substitutions.

While the mechanistic interpretations of resistance for HIV have been supported by structural data, no such data are available for HBV. Thus only homology models of the HBV RT, based on the HIV RT structure, have combined with genotypic and phenotypic data to provide mechanistic insight into HBV resistance. Consistent findings in the HBV model and HIV structure (e.g. lamivudine resistance, [18]) support the reliability of the HBV model, although it still lacks the validation of direct structural data. This remains an important challenge in the field of antiviral therapy for HBV.

## Materials and Methods

### Antiviral Compounds

ETV was prepared at BMS. The triphosphate form of ETV was prepared by TriLink Biotechnologies, Inc. (San Diego, CA), Other NRTIs and triphosphates were available from Moravek Biochemicals (Brea, CA) or TriLink.

### Cells and Viruses

HepG2 human hepatoma cells (American Type Culture Collection) were maintained as described [10]. The laboratory clone of HBV was the genotype D ayw serotype clone kindly provided by Dr. Steven Goff in the plasmid pCMV-HBV [41]. Plasmids expressing HBV RT with resistance substitutions were prepared using QuikChange® site-directed mutagenesis (Stratagene, LaJolla, CA).

### *In Vitro* HBV Polymerase Assay

To provide the data in Tables 1 & 2, intracellular HBV nucleocapsids were isolated from HBV transfected HepG2 cells and used in endogenous polymerase assays (EPA) [42], exactly as previously described in detail [5]. Essentially, clarified, detergent and nuclease-treated HBV-transfected cell lysates were centrifugally sedimented through a 25% wt/vol sucrose cushion. Sedimented nucleocapsids were used in polymerase reactions with [33P]-labeled dNTPs and TCA-precipitable radioactivity was used as an indication of the HBV polymerase activity, as described [5].

In order to increase the activity of the EPA reactions, we used nucleocapsids from cells treated with phosphonoformic acid (foscarnet, PFA; Sigma) during culture. PFA noncompetitively inhibits the polymerase of all hepadnaviruses studied, as well as other reverse-transcriptases and DNA polymerases (reviewed in Oberg, 1989 [43]). PFA has been shown to inhibit elongation by human HBV polymerase in vitro [19], [44], [45], and even HBV polymerase expressed from recombinant insect cells [19], [46] or in cell-free systems [47]. PFA inhibits synthesis of all HBV DNA forms and both strands of HBV DNA, but not priming, and has a cell culture EC_50_ of approximately 5 mM for HBV [48]. Therefore, we used 1 mM PFA in cultures, a level that would partially inhibit the elongation of HBV DNA within nucleocapsids, but still enable sufficient synthesis to quantify nucleocapsids using HBV real-time-PCR, as described [5].

The HBV polymerase dNTP K_m_ values were determined from similar assays where the concentration of dNTP was serially diluted from 1000 nM to 0.3 nM. All other dNTPs were used at a fixed concentration equal to the highest test dNTP concentration with one-tenth of the TTP in the form of [α^33^P] TTP (3000 Ci/mmol; PerkinElmer, Boston, MA). The apparent K_m_ values were obtained from Lineweaver-Burke plots of the data generated by GraphPad PRISM™ software version 3.0 (San Diego, CA). The K_i_ values were calculated using the Cheng-Prusoff equation: K_i_ = IC_50_/(1+[S]/K_m_) [49]. The enzyme concentration within HBV nucleocapsid preparations was found by determining the level of covalently-linked HBV DNA using quantitative real-time PCR [9] and converting to genome equivalents [1 pg double stranded HBV DNA = 3×10^5^ genome equivalents [34]]. The primers were positioned near the 5′ end of minus strand DNA in an attempt to measure all enzymatically active molecules.

### HBV Cell Culture Susceptibility

HepG2 cell culture susceptibility assays were performed in cells transfected with HBV expression plasmids and cultured in the presence of a titration of antiviral compounds, as described in detail elsewhere [7]. The levels of replicated, released HBV virions was measured at day five through immunocapture of detergent released nucleocapsids with anti-HBV core Ab and quantification of encapsidated HBV DNA [5]. Data were plotted as percent inhibition and EC_50_ values calculated as the drug concentration where 50% inhibition of extracellular HBV occurred.

### Metabolic Labeling of HBV DNA in Culture

The results in Figure 1 were derived from intracellular labeling of HBV DNA with [3H]-ETV, and quantitating relative to total HBV DNA. Cells were transfected and grown for two days in defined media (OptiMEM I; Invitrogen, Carlsbad, CA) containing either 100 nM [^3^H]-ETV (7.3 Ci/mmol) or [^3^H]-thymidine (80.2 Ci/mmol; PerkinElmer). Intracellular HBV nucleocapsids were isolated by sedimentation through sucrose, as described above. [^3^H]-ETV-labeled DNA was from cultures with [^3^H]-ETV. Total HBV DNA was determined on the same samples using quantitative real-time-PCR, as described [5]. An additional control for total HBV DNA used nucleocapsids isolated from parallel cultures labeled with [^3^H]-thymidine. Both methods gave similar results. Radiolabeled HBV DNA was quantified by mixing nucleocapsids with an equal volume of 20% ice-cold trichloroacetic acid, collected by vacuum filtration on GF/B plates (PerkinElmer) and analyzed for [^3^H] by scintillation counting of acid-insoluble material.

### HBV Cell Culture Strand-Specific EC_50_ Determination

Cell culture susceptibility assays were performed as above, except that extracted HBV DNA was blotted onto nylon membranes and hybridized with strand-specific riboprobes generated as described [4] except the riboprobes were fluorescein-labeled and a chemiluminescent signal was generated using an alkaline phosphatase-conjugated anti-fluorescein antibody as specified in the manufacturer's instructions (Amersham; Piscataway, NJ). Both sense and antisense riboprobes, which were 1168 bp in length and complementary to Xba I (nt 2143) to XhoI (nt 129) were generated by in vitro transcription (T3 and T7-Maxiscript; Ambion, TX). The double-stranded DNA probe used for comparison was the unit length 3.2-kbp HBV genotype D genome fragment.

### Homology Models of HBV-RT/DNA Complex

A homology model for the wildtype HBV-RT/DNA/dGTP complex was developed based on the sequence alignment between HBV RT and HIV RT [18] and the HIV-RT/DNA x-ray structure (1RTD.pdb) [50] using the Protein Design Module in Quanta (QUANTA Modeling Environment release 2000. Accelrys Software Inc., San Diego, CA.). Additional HBV-RT homology models were constructed with the LVD (M204V+L180M) and adefovir (A181T/V and N236T) resistance substitutions, and the ETVr signature substitutions T184G & S202I, or M250V were constructed on the LVDr (M204V+L180M) model. An additional model of the M250V was also constructed in WT HBV RT. The two HBV RT active site Magnesium ions and relative positions were homology built from the 1RTD.pdb structure. Note: as observed in our earlier study an extensive hydrogen-bonding network exists between HBV-RT and the tri-phosphate moiety [see figure 1B from [3]] of a NTP or equivalent inhibitor form. The relative position of the tri-phosphate moiety to the YMDD loop is further defined by the Mg ion bridge between the tri-phosphate moiety and D83/D205 which positions and anchors D205 of the YMDD loop and the tri-phosphate moiety of the NTP/inhibitor into the NTP binding site. Modeling NTP's or inhibitor's bound in the presence of the Mg ion bridge produces models with YMDD loop and NTP's/inhibitor's tri-phosphate conformations that were in close agreement with those observed in HIV RT x-ray structures, however, when modeled in the absence of the Mg ion bridge the structures are noticeably distorted (data not shown), highlighting the need to include the Mg ions in the model.

Using the DNA from the 1RTD.pdb structure as the template, different HBV RT models were constructed including the DNA [d(GCXCCGGCGCTCG)-d(CGAGCGCCGG)] and RNA/DNA [(GCXCCGGCGCTCG)-d(CGAGCGCCGG)] to model the four DNA base types and the nucleotide HBV-RT inhibitors (ETV (X = C), LVD (X = G), adefovir (X = T), and telbivudine (X = A)) bound in the dNTP binding site. Two additional RNA/DNA models were constructed with ETV modeled at the 3′(+1), and 3′(+2) positions of the newly synthesized DNA strand. The region around the WT HBV RT dNTP binding site was equilibrated, with dGTP bound in the dNTP binding site, by running a restrained 2 nanosecond molecular dynamics simulation.

Modeling studies were conducted with Quanta (QUANTA Modeling Environment release 2000. Accelrys Software Inc., San Diego, CA.) and CHARMM [51] running on a Silicon Graphics computer. The modeling figures presented were produced using DS ViewerPro 6.0 (Accelrys Software Inc., San Diego, CA.). The CHARMM parameters [52] were used for all calculation carried out within the QUANTA program while the CHARMM22 [53] and CHARMM27 parameters [54] were used for the molecular dynamics simulations. Switching functions were used in both energy minimization and molecular dynamics for the nonbonded, van der Waals and electrostatic interactions between 8 Å–10 Å with an 12 Å cutoff [55], [56]. The GBORN module [57] within CHARMM was used to calculate the Generalized Born salvation energy and forces. The Verlet algorithm [58] was used to calculate the classical equations of motion for the atoms and the X-H bonds were fixed using the SHAKE algorithm [59] during MD. The following restraints were applied during all calculations: All residues with one or more atoms within 13 Å of the dGTP bound in the dNTP site were not constrained; the residues within the 13 Å–20 Å zone from dGTP were positionally constrained with a 5 kcal harmonic tether, while all residues outside the 20 Å zone were tethered with a 50 kcal harmonic constraint. Each system was minimized with 200 steps of steepest descents followed by 500 steps of Adopted-Basis Newton Raphson minimization. The following molecular dynamics protocol was applied to each system: In a 1.5 pico-seconds (psec) heating phase the temperature was raised to 300 K in steps of 10 K over 0.05 psec blocks. The MD velocities were reassigned after every step based on the Gaussian approximation to the Maxwell Boltzmann distribution. This was followed by an equilibration phase in which the velocities were allowed to rescale over the next 18.5 psec in steps of 0.25 psec to stabilize the system within a 300±5 K window. The production phase continued for another 2 nsec where the velocities were allowed to rescale every 0.5 psec to keep the system within a 300±10 K window.
